# Multipotent Genetic Suppression of Retrotransposon-Induced Mutations by *Nxf1* through Fine-Tuning of Alternative Splicing

**DOI:** 10.1371/journal.pgen.1000484

**Published:** 2009-05-15

**Authors:** Dorothy Concepcion, Lisbeth Flores-García, Bruce A. Hamilton

**Affiliations:** 1 Department of Medicine, University of California San Diego School of Medicine, La Jolla, California, United States of America; 2 Department of Cellular and Molecular Medicine, University of California San Diego School of Medicine, La Jolla, California, United States of America; 3 Rebecca and John Moores UCSD Cancer Center, University of California San Diego School of Medicine, La Jolla, California, United States of America; The Jackson Laboratory, United States of America

## Abstract

Cellular gene expression machinery has coevolved with molecular parasites, such as viruses and transposons, which rely on host cells for their expression and reproduction. We previously reported that a wild-derived allele of mouse *Nxf1* (Tap), a key component of the host mRNA nuclear export machinery, suppresses two endogenous retrovirus-induced mutations and shows suggestive evidence of positive selection. Here we show that *Nxf1^CAST^* suppresses a specific and frequent class of intracisternal A particle (IAP)-induced mutations, including *Ap3d1^mh2J^*, a model for Hermansky-Pudlak syndrome, and *Atcay^hes^*, an orthologous gene model for Cayman ataxia, among others. The molecular phenotype of suppression includes ∼two-fold increase in the level of correctly-spliced mRNA and a decrease in mutant-specific, alternatively-processed RNA accumulating from the inserted allele. Insertional mutations involving ETn and LINE elements are not suppressed, demonstrating a high degree of specificity to this suppression mechanism. These results implicate Nxf1 in some instances of pre-mRNA processing, demonstrate the useful range of *Nxf1^CAST^* alleles for manipulating existing mouse models of disease, and specifically imply a low functional threshold for therapeutic benefit in Cayman ataxia.

## Author Summary

Retroviruses and transposable elements are molecular parasites that integrate into the host genome and require host cell machinery for gene expression, replication and dissemination. Integrating elements can alter the expression of nearby host genes through both transcriptional and post-transcriptional mechanisms. Components of the host cell machinery that can adapt to favor genetic programs of the host cell over those of the parasite may afford one level of innate immunity. In laboratory mice, endogenous retroviruses are virus-derived mobile elements that account for many spontaneous mutations. A frequent class involves retrotransposition into introns of genes in the transcriptional sense orientation, which alters host gene pre-mRNA splicing. Here we show that for the intracisternal A particle (IAP) family of endogenous retroviruses, an allele of the canonical mRNA export factor Nxf1 found in wild Asiatic mice (*Mus musculus castaneus*) suppresses most insertions of this class (six of seven tested). To our knowledge, these results make *Nxf1* the most broadly interacting modifier gene yet documented in this well-studied species. These results have significant implications for manipulating gene expression in mouse models of disease, the role of Nxf1 in pre-mRNA processing and in the dynamic range for therapeutic intervention in Cayman ataxia.

## Introduction

Retroviruses and transposable elements both utilize host cell factors for their own expression and influence the expression of adjacent host genes through a variety of mechanisms. Components of host cell gene regulatory machinery that interact with molecular parasites may be regarded as components of innate immunity if they can discriminate between host and parasite expression [1]. The generality and exploitability of any given mechanism is an important practical question. Nuclear-cytoplasmic export of RNA is an important point of contact between molecular parasites and host genomes that may fit this criterion for several molecular parasites in mice and humans [2],[3]. We have previously reported that a wild-derived allele of *Nxf1*, which encodes the major mRNA nuclear export factor, can significantly suppress two mutations caused by insertions of endogenous retroviruses into introns of cellular genes by modulating their mature transcript levels ∼2 fold [4]. A 16 kb transgene containing the full *Nxf1* haplotype, but no other recognized gene, was able to confer the modifier phenotype. Whether this interaction could be generalized to a broader class of insertional events, and if so for what range of insertions, was limited by the relatively small number of events examined.

Nxf1 (also called Tap) was first described as a cellular factor that interacts with the Tip protein of herpesvirus saimiri [5] and subsequently shown to be an essential host factor for nuclear export of unspliced viral genomes of simple retroviruses [6]. Although recruitment of Nxf1 to cellular mRNPs may generally be mediated by protein contacts [7],[8], both Nxf1 and its yeast homolog Mex67p also bind RNA directly [9]–[11]. In mammals, known direct targets of Nxf1 include both exogenous and endogenous viral RNAs as well as host sequences [6], [12]–[14]. In addition, we previously reported that one *Nxf1* haplotype shows hallmarks of recent positive selection in wild *Mus musculus castaneus* accessions [4], which may suggest a host-pathogen interaction mediated by Nxf1 in wild populations.

Endogenous retroviruses (ERVs) are non-infectious molecular parasites that are frequent mutagens in mice. Several families of ERV are highly polymorphic among classical inbred strains and among wild accessions [15]. In laboratory mice, ERV insertions account for 10–15% of spontaneous mutations [16],[17], depending on the strains from which estimates are drawn. The intracisternal A particle (IAP) and MusD/early transposon (ETn) families of ERV, which account for most of these, have different apparent rates of transposition in different inbred strains: IAPs appear to be particularly active in C3H strains and ETn elements in A strains [16]. Characteristics of autonomously active copies have been described [18]. Interestingly, the size distribution for newly integrated ETn elements is both broader and, on average, a lower percentage of full length than for IAP elements [19]. As both families are thought to have derived originally from infectious viruses, mechanisms that regulate ERVs or mitigate their impact on host genomes may have broader implications for both gene expression and host-parasite interactions.

To test the range of insertion events for which the modifier activity of *Nxf1^CAST^* is effective, we examined gene expression, visible phenotypes, or both for five additional IAP, one LINE, and seven ETn insertion alleles. The host genes cover a wide range of phenotypes, expression patterns, and biochemical pathways:

- The genes mutated in classical coat color mutations *mahogany* (*Atrn*) and *mahoganoid* (*Mgrn*) both mediate intercellular signaling by secreted agouti protein. *Atrn* encodes a transmembrane accessory receptor [20],[21], while *Mgrn* encodes an E3 ubiquitin-protein ligase that participates in endosomal trafficking [22]. Spontaneous alleles at either gene range in effect from modest coat color changes to spongiform neurodegeneration with associated neurological deficits [20],[21],[23],[24]. Among these alleles, *Atrn^mgL^* and *Mgrn^md^* are de novo IAP insertions into introns in the transcriptional sense orientation [24],[25] that decrease the steady-state level of correctly processed mRNA in mutant tissues, resulting in moderate coat color darkening, but lacking the neurodegeneration seen in stronger alleles.
- Spontaneous *mocha* alleles of the intracellular trafficking adapter protein gene *Ap3d1* [26] include a hypomorphic IAP insertion allele (*mh^2J^*) that reduces levels of wild-type RNA and protein. In addition to coat color dilution caused by sorting defects in melanosomes, *mh^2J^* and more severe alleles show substantial mortality, neurological and behavioral impairments [27]. Because mutations in other Ap3 complex proteins are associated with Hermansky-Pudlak syndrome, *mocha* mice have been used to model this disease [26],[27].
- The *ataxia^J^* mutation is an IAP insertion into an intron of the ubiquitin specific protease gene *Usp14*. Although the protein targets have not been systematically identified, loss of Usp14 activity results in synaptic defects that manifest behaviorally as tremor and ataxic gait in *Usp14^axJ^* mice [28].
- The classical mouse locus *jittery* is orthologous to the CRAL-TRIO domain gene *ATCAY* mutated in human Cayman ataxia [29]. Patients with this recessive disorder have a prominent but non-progressive psychomotor impairment consistent with cerebellar disease [30]. The *hesitant* mutation (*Atcay^hes^* ) is an IAP insertion into the first intron, resulting in profound locomotor deficits with no obvious neuroanatomical correlates [29].
- Mutations of the *Mitf* transcription factor gene block melanocyte development, causing white-spotting and other defects in mice and Waardenburg syndrome in humans. The mouse *black-eyed white* allele is a L1 LINE element inserted into an intron that disrupts splicing of one alternative 5′ exon [31]. Loss of this isoform results in recessive severe white spotting, such that the fur is most often completely white, with pigmented patches occurring in some animals. Weaker alleles of *Mitf* show larger and more frequent area of pigmented fur, providing a sensitive phenotypic readout for allele strength and modifier genes [32].
- The MusD/ETn family are endogenous retroviruses that are more closely related to the IAP superfamily than most other currently active mouse retroelements [33],[34]. BALB/cJ and A/J strains carry several recent MusD/ETn family insertions that are mutagenic with respect to host genes [19]. In particular, *Zhx2* is a transcriptional repressor required to down-regulate expression of *Afp* fetal globin RNA. Loss of *Zhx2* expression in BALB/cJ (but not other BALB/c lines) due to an ETn-II insertion results in persistent *Afp* expression into adulthood [35],[36]. Insertion of an ETn in the gene encodng dysferlin, *Dysf*, in A/J mice results in loss of expression and creates an orthologous gene model for human limb-girdle muscular dystrophy 2B [37].

Here we show that *Nxf1^CAST^* suppresses six of six IAP insertions of the IΔ1 class [38], the most frequent class of new insertions, but does not suppress a full-length IAP, a L1-LINE, nor any of six ETn insertion mutations. We quantify RNA and protein levels to show a consistent ∼2-fold increase in normal gene expression from the mutant allele in each case of suppression. Concomitant decrease in the expression of mutant-specific RNAs implicates Nxf1 in pre-mRNA processing in addition to its known role in mRNA export. For disease models and other mouse mutations induced by IAP-IΔ1 retrotransposition, *Nxf1^CAST^* provides a genetic rheostat for gene activity in situ.

## Results

### *Nxf1^CAST^* Suppresses RNA Expression Phenotypes of *Mgrn^md^*, but Not *Atrn^mgL^*

To test whether *Nxf1^CAST^* can suppress the RNA processing defects in *Atrn^mgL^* and *Mgrn^md^*, we examined whole brain RNA of progeny from genetic crosses to *Nxf1^CAST^*, comparing homozygous mutant littermates that differ in *Nxf1* genotype. Because each of these crosses also segregated other loci contributing to coat color, we did not assess pigmentation phenotypes for these two mutants.

**Figure 1:**
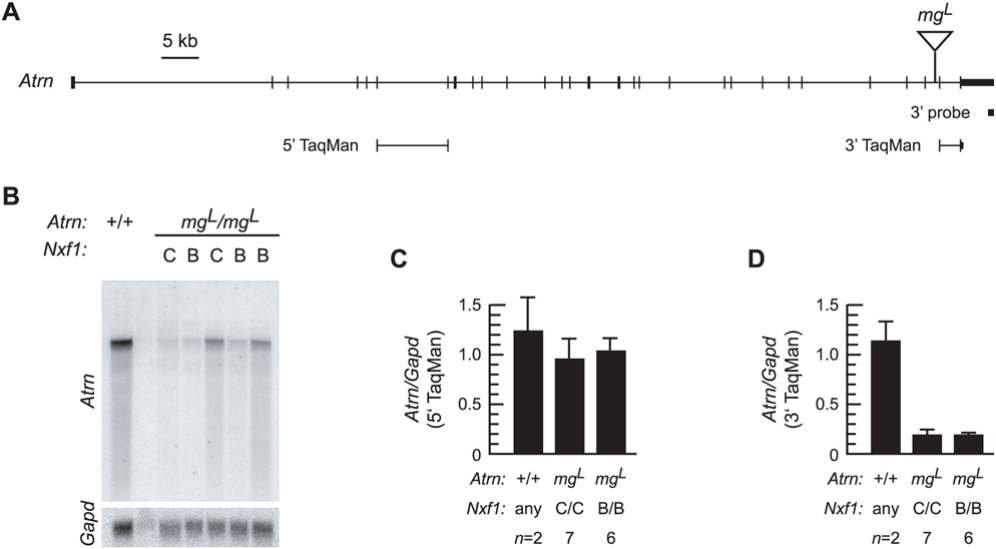
*Nxf1^CAST^* does not suppress mRNA deficit in *Atrn^mgL^*. (A) Scale diagram of the mouse *Atrn* locus indicates the location and approximate size of the IAP insertion in *mg^L^* allele and the Northern blot probe. (B) Northern blot analysis of brain poly(A)^+^ RNA (5 µg per lane) from non-mutant and mutant littermates shows reduced level of full-length *Atrn* mRNA in *mg^L^* animals, independent of *Nxf1* allele (B for C57BL/6, C for CAST/Ei). (C, D) Quantitative PCR (TaqMan) data shows nominal reduction in the abundance of spliced 5′ sequences, but ∼6-fold reduction of 3′ sequences, consistent with alternative splicing and 3′ end formation in the insertion, with no difference between *Nxf1* genotypes. Error bars indicate standard deviation.

For *Atrn* (Figure 1), abnormally processed message from *mg^L^* alleles are detected on Northern blots by probes containing exons 5′ to the insertion site, but not by the 3′ untranslated region ([25] and Figure 1A, B). Because the large but low-abundance normally spliced message was difficult to quantify reliably from Northern blots, we used TaqMan quantitative RT-PCR to assay RNA abundance in *mg^L^* mutant brains. Comparing *mg^L^* to control animals shows non-significant reduction in abundance of 5′ sequences (Figure 1C), but ∼6-fold loss of full-length transcript, represented by an assay 3′ to the *mg^L^* insertion (Figure 1D). However, this assay shows no effect of *Nxf1* genotype on *Atrn* expression.

**Figure 2 pgen-1000484-g002:**
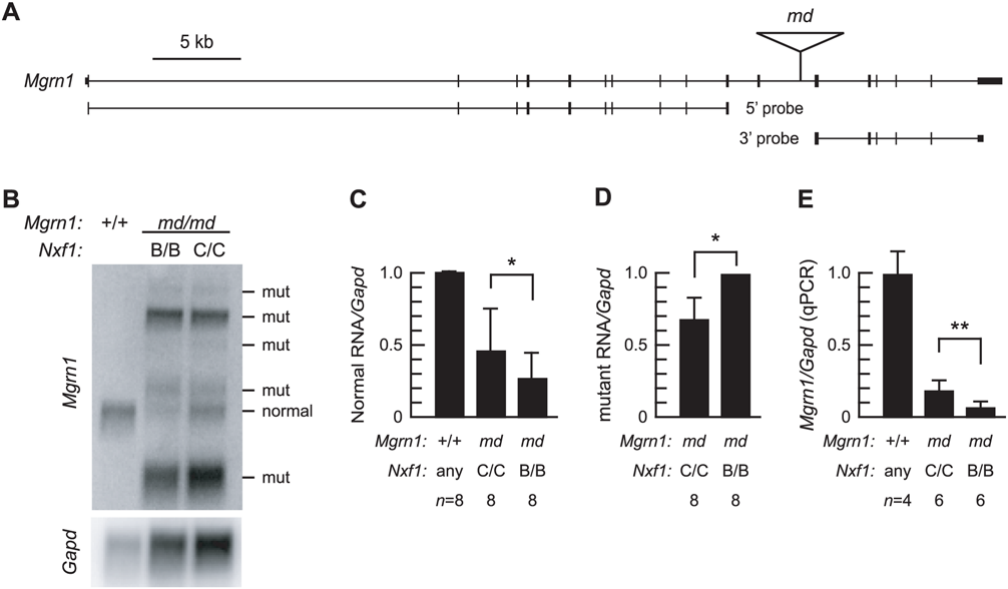
*Nxf1^CAST^* suppresses pre-mRNA processing defects in *Mgrn^md^*. (A) Diagram of the *Mgrn* locus indicates locations and sizes of the *md* IAP insertion and probes. (B) Northern blot of brain poly(A)^+^ RNA (3 µg per lane) shows increased level of full-length *Mgrn* RNA and concomitantly reduced levels of mutant-specific transcripts in *md* mutant mice in the presence of *Nxf1^CAST^*. (C, D) Quantification of multiple independent Northern blot experiments is shown. Graphs show means of replicate experiments normalized to nonmutant control samples on each blot. Error bars indicate standard deviation. Presence of the lowest molecular weight band was inconsistent across experiments and not included in panel D. *p≤0.05, Wilcoxon signed-ranks test applied to paired (same-blot) samples. (E) Quantitative RT-PCR across the inserted intron indicates ∼2-fold increase in correctly spliced *Mgrn1* RNA in the presence of *Nxf1^CAST^*. **p≤0.005, unpaired t-test with one tail.

In contrast, for *Mgrn*, *Nxf1*-dependent differences in the level of correctly and alternatively spliced RNA isoforms from *md* alleles were readily quantified (Figure 2). A probe 5′ to the *md* insertion (Figure 2A) detects both normal and mutant-specific *Mgrn* RNAs (Figure 2B). Correctly processed normal RNA is elevated in the presence of *Nxf1^CAST^*, while levels of several mutant-specific transcripts is decreased (Figure 2B–D), consistent with the mode of suppression previously reported for *Pitpn^vb^* and *Eya1^BOR^*. A probe 3′ to the insertion detects only the correctly spliced form, at levels comparable to the 5′ probe (not shown). Quantitative RT-PCR across the inserted intron confirms a ∼2-fold increase in correctly-spliced transcript levels by *Nxf1^CAST^* (Figure 2E).

### *Nxf1^CAST^* Suppresses RNA, Protein, and Phenotypic Expression of *Ap3d1^mh2J^*

**Figure 3 pgen-1000484-g003:**
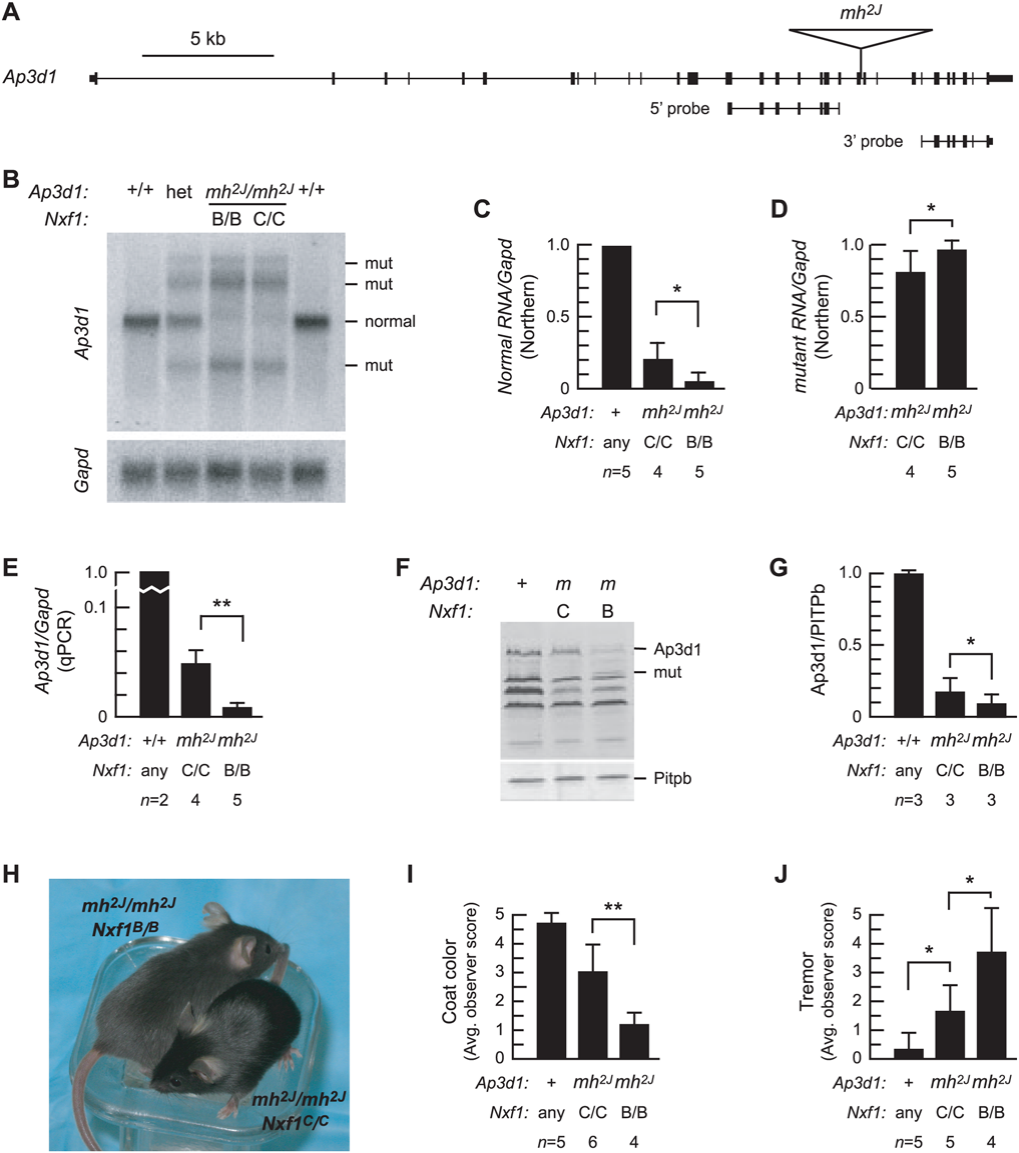
*Nxf1^CAST^* suppresses *Ap3d1^mh2J^* RNA, protein and visible phenotypes. (A) Diagram of *Ap3d1* shows location and size of the *mh^2J^* IAP insertion and Northern blot probes. (B) Northern blot of brain poly(A)^+^ RNA (5 µg per lane) shows that the level of full-length *Ap3d1* RNA in *mh^2J^* mice is partially restored and levels of mutant-specific RNAs are reduced in the presence of *Nxf1^CAST^*. (C) Quantification of Northern blot experiments with paired samples, showing means and standard deviation of normalized values across all experiments. *p≤0.05, Wilcoxon signed-ranks test using 5 pairs (using one *Nxf1* heterozygous sample to complete a pair) and *p*≤0.01, paired t-test with 4 pairs; each test has one tail. (D) Levels of mutant-specific RNAs appear decreased in the presence of *Nxf1^CAST^*, *p≤0.05, Wilcoxon signed-ranks test. (E) Quantitative RT-PCR analysis with primers spanning the inserted intron. **p = 0.0003, unpaired t-test with one tail. (F) Western blot of brain protein extracts shows increased level of Ap3d protein from *mh^2J^* in *Nxf1^CAST^* mice. (G) Quantification of replicate Western blots shows ∼2-fold increase in Ap3d with *Nxf1^CAST^*. *p = 0.03, paired t-test, one tail. (H) *mh^2J^* coat color dilution is attenuated in *Nxf1^CAST^*. (*I*) Average coat color scores, comparing mice to a printed grading matrix, and (J) tremor severity scores were assessed by observers blinded to genotype. Error bars indicate standard deviation. **p≤0.01, *p≤0.05, t-test with one tail.

To test *Nxf1^CAST^* activity on a mutation for which protein level and phenotype were accessible, we analyzed RNA and protein levels, coat color (eumelanin) dilution and tremor severity of *Ap3d1^mh2J^* mutant animals (Figure 3). Locations of the *mh^2J^* insertion and probes are indicated in Figure 3A. Although Northern blots show high variance between experiments, comparisons between paired subjects examined on each blot shows a statistically significant increase in normal-sized *Ap3d1* transcript and a modest decrease in mutant-specific transcript in the presence of *Nxf1^CAST^* (Figure 3B–D). Quantitative RT-PCR confirms the increase in correctly spliced RNA (Figure 3E). Western blots show a corresponding increase in full-length Ap3d1 protein levels detected by an antibody to N-terminal residues (Figure 3F,G). Correspondingly, a smaller protein species detected only in mutant animals is decreased in *Nxf1^CAST^* animals. As predicted from this molecular analysis, *Ap3d1^mh2J^* mutant animals also had improved pigmentation and neurological assessment scores in the presence of *Nxf1^CAST^* as rated by observers blinded to genotype (Figure 3H–J).

### *Nxf1^CAST^* Suppresses RNA, Protein, and Phenotypic Expression of *Usp14^axJ^*

**Figure 4 pgen-1000484-g004:**
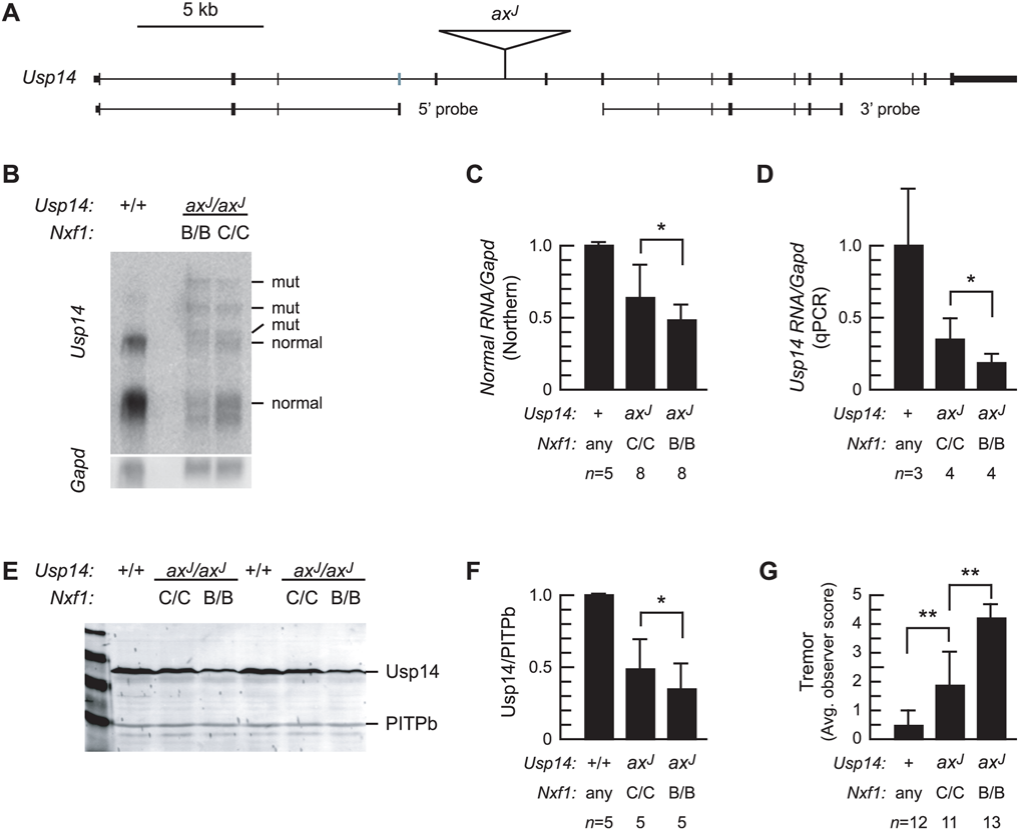
*Nxf1^CAST^* suppresses *Usp14^axJ^* expression and behavioral phenotypes. (A) Diagram of *Usp14* locus shows locations of the *ax^J^* IAP insertion and Northern blot probes. (B) Northern blot of brain poly(A)^+^ RNA (4 µg per lane) shows that the level of full-length *Usp14* RNA in *ax^J^* mice is partially restored and levels of mutant-specific RNAs are reduced in the presence of *Nxf1^CAST^*. (C) Quantification of paired samples from multiple Northern blot experiments, showing means and standard deviations. *p≤0.05, Wilcoxon signed-ranks test. (D) Quantitative RT-PCR shows ∼2-fold difference in expression of correctly spliced *Usp14* RNA in mutant brains homozygous for *Nxf1^CAST^*. *p≤0.05, unpaired t-test with one tail. (E) Western blot of brain protein extracts shows increased level of Usp14 protein from *ax^J^* in *Nxf1^CAST^* mice. *p≤0.05, Wilcoxon signed-ranks. (F) Quantification of replicate Western blots shows increased Usp14 expression in the presence of *Nxf1^CAST^*. *p≤0.05, Wilcoxon signed-ranks. (G) Neurological assessment scores for tremor assigned by observers blinded to genotype show highly significant improvement in animals homozygous for *Nxf1^CAST^*. Error bars indicate standard deviation. **p<0.01 unpaired t-test. See Videos S1 and S2.

We similarly tested *Nxf1^CAST^* activity on molecular and visible phenotypes of *Usp14^axJ^* (Figure 4). The insertion and probes used are indicated in Figure 4A. Quantification of Northern blots and RT-PCR experiments from brain RNA shows significantly increased levels of correctly processed RNA in the presence of *Nxf1^CAST^* (Figure 4B–D). Quantification of Western blots shows that this is translated into an increased level of Usp14 protein (Figure 4E,F). Behaviorally, *Usp14^axJ^* mutant animals also showed improved neurological assessment scores, with visibly reduced tremor amplitude in the presence of *Nxf1^CAST^* (Figure 4G and Videos S1 and S2). In contrast to other mutations suppressed by *Nxf1^CAST^*, normalized levels of mutant-specific isoforms of *Usp14* RNA did not differ significantly by *Nxf1* genotype. Comparing Northern blots hybridized with either 5′ or 3′ probes (as indicated in Figures 2–5), we find *Usp14^axJ^* and *Eya1^BOR^* differ from other suppressed mutations in producing RNA isoforms that contain 5′ exons, IAP sequences and 3′ exons [4],[28] where most others produce primarily 5′ exons and terminal IAP sequences.

**Figure 5:**
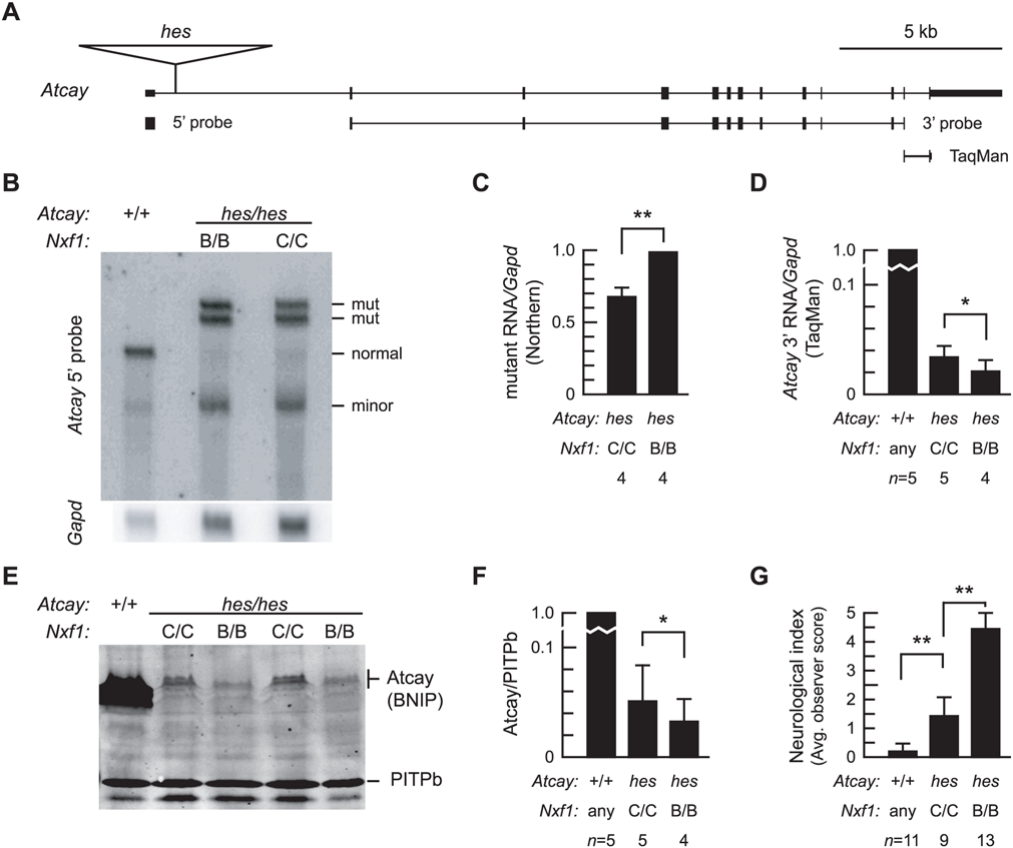
*Nxf1^CAST^* suppresses *Atcay^hes^* RNA, protein and behavioral phenotypes. (A) Diagram of *Atcay* shows location of the *hes* IAP insertion, Northern blot probes and TaqMan assay. (B) Northern blot of brain poly(A)^+^ RNA (8 µg per lane) shows that level of mutant-specific *Atcay* transcripts in *hes* mice are reduced in the presence of *Nxf1^CAST^*. (C) Quantification of replicate Northern blot experiments shows reduced level of mutant-specific transcripts in *Nxf1^CAST^* brains. **p<0.01 paired t-test with one tail. (D) Quantitative PCR (TaqMan) analysis of *Atcay* RNA in *hes* homozygotes shows ∼2-fold increase in *Nxf1^CAST^*. *p<0.05 paired t-test with one tail. (E) Western blot of brain protein extracts shows increased level of Caytaxin protein from expression of *Atcay* in *Nxf1^CAST^* mice. (F) Quantification of replicate Western blots shows ∼2-fold increase in Caytaxin with *Nxf1^CAST^*. *p<0.05, t-test, one tail. (G) Average neurological assessment scores assigned by observers blinded to genotype show highly significant improvement in animals homozygous for *Nxf1^CAST^*. Error bars indicate standard deviation. **p<0.01, t-test, one tail. See Videos S3 and S4.

### *Nxf1^CAST^* Suppression of *Atcay^hes^* Indicates High Sensitivity of Phenotype to Increased Expression

To test *Nxf1^CAST^* activity in the context of a human disease model, we analyzed several levels of molecular and behavioral phenotypes for the *Atcay^hes^* mutation (Figure 5). The locations of the *hes* insertion and probes are indicated in Figure 5A. *Atcay^hes^* alleles express prominent mutant-specific *Atcay* RNAs and very low levels of correctly processed full-length RNA [29]. Northern blots to quantify size-specific RNA levels show reduced level of each mutant-specific RNA detected by a probe 5′ to the insertion (Figure 5B,C). A probe 3′ to the insertion detects only the full length “normal” RNA and is quantifiable only in non-mutant samples (not shown). To measure levels of normal RNA in mutant samples, we used a quantitative RT-PCR (TaqMan) assay (Figure 5D). The presence of *Nxf1^CAST^* significantly increases the level of correctly processed *Atcay* RNA accumulating from *hes* alleles. This difference is also translated into higher levels of the encoded Caytaxin/BNIP-H protein (Figure 5E,F). *Atcay^hes^* mutant animals have profound ataxia and an unusual jumping behavior (see Video S3). *Nxf1* genotype had a highly significant impact on *Atcay^hes^* neurological phenotypes as rated by multiple observers blinded to genotype, including both reduced ataxia and complete elimination of jumps from open field behavior (Figure 5G and Videos S3 and S4).

### *Nxf1^CAST^* Does Not Suppress L1-LINE Mutation of *Mitf^mi-bw^*

To test a non-viral class of retrotransposon, we examined whether *Nxf1^CAST^* would suppress the *black-eyed white* L1-LINE insertion allele of *Mitf*. This mutation results in loss of pigmented melanocytes and extreme white spotting, leaving only occasional patches of pigment on the head or ears. Despite this low threshold for phenotype modulation, and known effects of other strain backgrounds, we saw no evidence for modification by *Nxf1^CAST^* in an F2 cross. Among 14 *Mitf^mi-bw^*, *Nxf1^B6^* and 9 *Mitf^mi-bw^*, *Nxf1^CAST^* doubly homozygous progeny, we observed a single animal of each genotype with dark patches on the head or ears.

### *Nxf1^CAST^* Does Not Suppress Typical ETn Insertions

**Figure 6:**
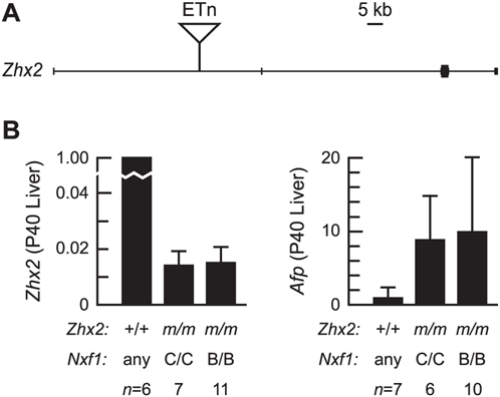
*Nxf1^CAST^* does not suppress ETn-induced *Zhx2^Afr^* mutation. (A) Diagram shows genomic organization of *Zhx2*, including location of the ETn-IIa insertion. Insertion is not to scale. (B) Quantitative RT-PCR shows reduced *Zhx2* expression from mutant alleles (*m*), but no suppression by *Nxf1^CAST^*. Persistent *Afp* expression in adult liver in mutant animals is highly variable among F2 animals at P40, but not significantly different between *Nxf1* genotypes. Error bars indicate standard deviation.

We tested *Nxf1^CAST^* activity on both sense and antisense-oriented ETn insertions of recent origin in both BALB/cJ and A/J mice. Expression levels of *Zhx2* and its repression target *Afp* were assayed by quantitative RT-PCR from adult liver at P40 from 24 BALB/cJ x B6-*Nxf1^CAST^* F2 animals selected by genotype (Figure 6A,B). The BALB/cJ-derived insertion allele expressed ∼1.5% non-mutant levels of *Zhx2*, with no difference between *Nxf1* alleles. Similarly, the effect on *Afp* persistence, potentially a more sensitive indicator of *Zhx2* function, showed no significant difference between *Nxf1* alleles, although inter-individual variation was high (Figure 6B, right panel), likely due to other factors segregating in this cross [39].

**Figure 7:**
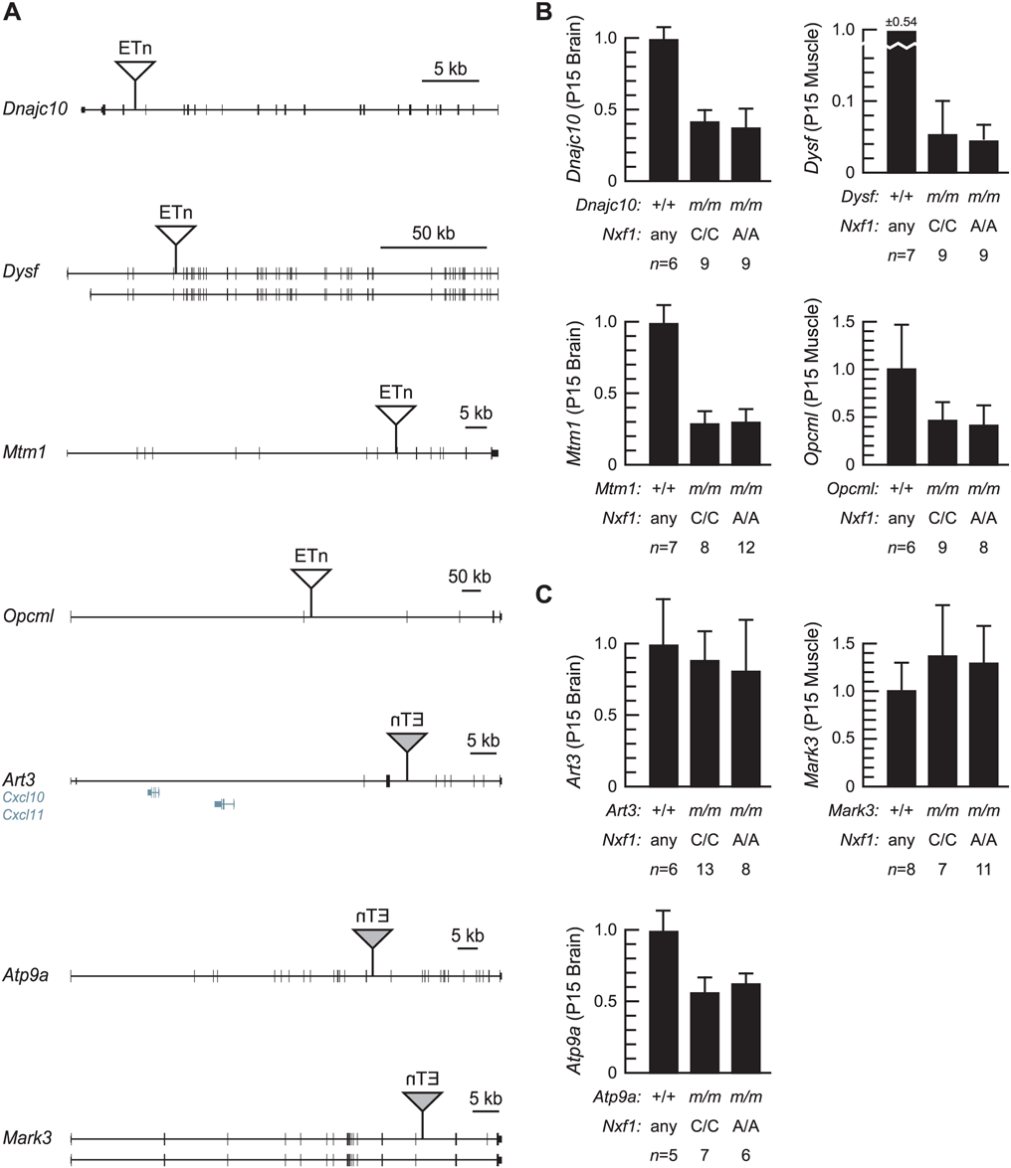
*Nxf1^CAST^* does not suppress ETn-induced mutations in A/J. (A) Genomic organization of five sense and three antisense-oriented ETn insertions monitored in F2 mice from A/J x B6–*Nxf1^CAST^*. Insertions are not to scale. (B) Quantitative PCR (SYBR green) on brain and/or muscle cDNA shows reduced expression of sense-oriented ETn alleles, but no significant differences between *Nxf1* genotypes. *m*, insertion allele at each indicated locus. (C) Quantitative PCR shows modest (*Atp9a*) or no difference (*Art3*, *Mark3*) in expression of antisense-oriented ETn insertions, with no difference attributable to *Nxf1*. Error bars indicate standard deviation.

We tested the ability of *Nxf1* to elevate transcript levels for another 5 sense and 3 antisense intronic ETn insertions in a second cross, A/J x B6-*Nxf1^CAST^* (Figure 7). Genomic organization and the location and orientation of the insertions are indicated (Figure 7A). Quantitative RT-PCR measurements from brain or muscle (depending on known pattern of expression for each gene) showed no significant differences between *Nxf1* genotypes for either sense or antisense insertions (Figure 7B,C). A fifth sense-oriented insertion, in *Prkca*, showed no difference between inserted and uninserted alleles for either RNA or protein levels in this cross.

### *Nxf1^CAST^*-Sensitive Insertions Carry the IΔ1 Deletion

**Figure 8:**
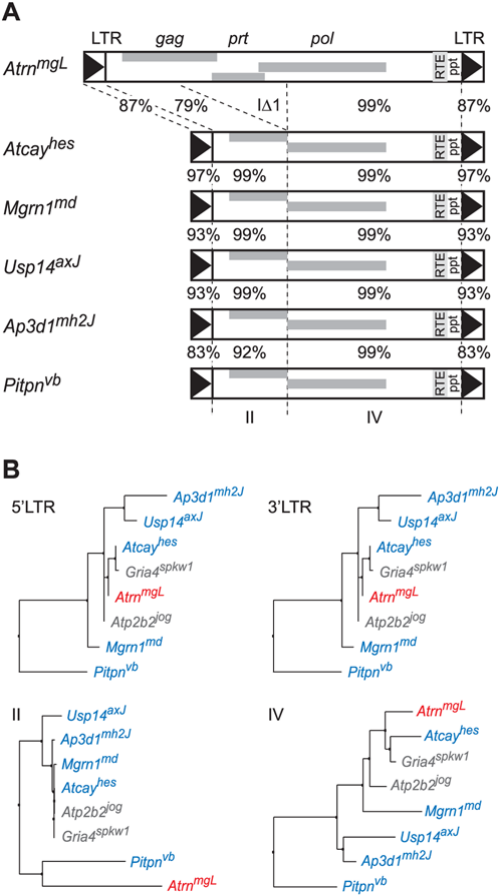
The *gag* region and IΔ1 deletion differentiate *Atrn^mgL^* from other IAP insertions. (A) Organization and pairwise percent identity of sequenced IAP elements from mutations in this study. RTE, RNA transport element; ppt, polypurine tract. Sequences have been deposited in GenBank (Accession numbers FJ854355–FJ854360). (B) Neighbor-joining trees for each of four aligned sequence blocks show similar topology for LTR and *pol*/RTE/ppt regions. For the undeleted portion of the *gag* gene, *Atrn^mgL^* clusters with *Pitpn^vb^* as a separate group. Recently described and sequenced IAP-IΔ1 elements inserted in *Atp2b2^jog^* and *Gria4^spkw1^* mutations fall within the group of suppressed elements in all four sequence regions.

Among sense-oriented IAP elements, only *Atrn^mgL^* was not suppressed by *Nxf1^CAST^*; as the inserted intron does not appear to be differentiated in position, length, or sequence composition from mutations that were suppressed (Figures 1–5 and data not shown) we determined the DNA sequence of each of these inserted elements, as well as the original *Pitpn^vb^* insertion [4],[40]. We amplified each insertion using high-fidelity PCR optimized for long sequences, using unique primers flanking each insertion site (Supplemental material online). *Ap3d1^mh2J^*, *Atcay^hes^*, *Mgrn1^md^*, *Pitpn^vb^* and *Usp14^axJ^* insertions all amplified fragments of 5.5 to 6.0 kb, while the *Atrn^mgL^* insertion required modified conditions to support adequate amplification of a unique ∼8 kb product. DNA sequence analysis showed that the *Atrn^mgL^* element is a full length (type I) IAP, while each of *Nxf1^CAST^*-sensitive elements includes the 1.9 kb deletion of *gag-pol* sequence typical of type IΔ1 elements [38] (Figure 8A). All 6 elements belong to the IAPEz subfamily (www.repeatmasker.org), and contain an RTE-D transport element [41],[42] near the 3′ LTR. Calculated trees for each segment of aligned sequence shows that the full length *Atrn^mgL^* element is not otherwise an outlier in overall sequence composition, except for the undeleted region of the *gag* gene (Figure 8B). Inclusion in the tree of two recently identified IAP-IΔ1 insertions, *Atp2b2^jog^* and *Gria4^spkw1^* [43],[44], suggests that they too should be sensitive to *Nxf1^CAST^*-mediated suppression as they fall within sequence clades of suppressed elements for each segment.

## Discussion

Our results show that *Nxf1^CAST^* suppresses a broad and frequent class of IAP-induced mutations. The magnitude of increased normal transcript is ∼2-fold and the impact on gene expression and behavioral phenotypes are significant in each case of this class examined. *Nxf1^CAST^* increases the steady-state level of correctly spliced host gene transcript and almost always decreases the level of mutant-specific alternatively spliced transcript for six of seven sense-oriented IAP insertions examined to date (Table 1). The one exception, *Atrn^mgL^*, differs from all of the suppressed elements we sequenced in having an intact *gag-prt-pol* coding sequence. Sequences within the deleted region may therefore mediate an additional level of *Atrn* repression that is not relieved by *Nxf1^CAST^*. Each insertion, including *Atrn^mgL^* also had a number of more subtle sequence variations, including smaller indels and further studies will be required to clarify which sequence differences contribute to the lack of suppression. However, the current data do provide a clear guide for the class of insertional mutation most likely to be quantitatively modulated by *Nxf1^CAST^*, type IΔ1 IAPs, which are by far the most frequent class recovered from spontaneous mouse mutations. While it is possible that other genes within the congenic interval contribute to any one effect, transgenic mouse and lentiviral gene transfer studies with *Pitpn^vb^* indicate that the main effect is due to *Nxf1*, as do the consistency of findings across all six suppressed mutations. Negative data from six ETn-inserted loci indicate that *Nxf1^CAST^* is highly selective, and therefore unlikely to result in collateral changes in gene expression when used to manipulate IAP-induced mutations. Indeed, preliminary microarray data failed to identify any significant expression changes in whole brain RNA (B.A.H., unpublished data).

**Table 1:** Summary of genetic crosses to test *Nxf1^CAST^* modifier effects.

Mutation	Insertion	Orientation	Increase normal transcript level?	Decrease mutant transcript levels?	Elevate protein level?	Suppress phenotype?	Ref.
*Pitpn^vb^*	IAP	sense	yes	yes	yes	yes	[4]
*Eya1^BOR^*	IAP	sense	yes	yes		yes	[4]
*Agouti^iy^*	IAP	antisense				no	[4]
*Axin^Fu^*	IAP	antisense				no	[4]
*Hairless^hr^*	MuLV	sense				no	[4]
*Myo5a^d^*	MuLV	sense				no	[4]
*Agouti^a^*	VL30	antisense				no	[4]
*Ap3d1^mh2J^*	IAP	sense	yes	yes	yes	yes	This work
*Atcay^hes^*	IAP	sense	yes	yes	yes	yes	This work
*Atrn^mgL^*	IAP	sense	no			no	This work
*Mgrn1^md^*	IAP	sense	yes	yes			This work
*Usp14^axJ^*	IAP	sense	yes	yes	yes	yes	This work
*Mitf^mi-bw^*	L1-LINE	sense				no	This work
*Zhx2^Afrb^*	ETn	sense	no			no	This work
*Dnajc10^AJ^*	ETn	sense	no				This work
*Dysf^prmd^*	ETn	sense	no				This work
*Mtm1^AJ^*	ETn	sense	no				This work
*Opcml^AJ^*	ETn	sense	no				This work
*Prkca^AJ^*	ETn	sense	n/a				This work
*Art3^AJ^*	ETn	antisense	n/a				This work
*Atp9a^AJ^*	ETn	antisense	no				This work
*Mark3*	ETn	antisense	n/a				This work

The simplest explanation for the molecular data from the six mutations suppressed by *Nxf1^CAST^* would be for Nxf1 to participate in pre-mRNA processing prior to the completion of splicing. This could occur by recruitment of Nxf1 to the nascent transcript by sequences in the IAP (or proteins bound to them co-transcriptionally) and subsequent interactions between Nxf1 and other components of the mRNP. Under such a model, amino acid differences (S48P and E610G) between the allelic Nxf1 proteins would alter the balance of alternative splicing either directly through interactions with splicing machinery or indirectly through an effect on transcriptional elongation rate or preference for termination site in the insertion. An alternative explanation might be for the export activity of Nxf1 to drive the nascent RNP into a territory with different relative activities for splicing and degradation, but this seems more difficult to reconcile with simultaneously increased levels of the correctly spliced message and decreased levels of the mutant splice form in five of the six suppression events.

Nxf1 protein interacts with several factors that could influence alternative splicing, including U2AF35 [45], several SR proteins [7],[8],[46],[47], and components of the TREX complex [48],[49]. Nxf1 is also recruited to the class of retroviral RNA transport elements (RTE-D), found in the IAPs we sequenced from suppressed mutations, through its interaction with RBM15 (OTT1) [42], which has also been linked to both splicing and export of Epstein-Barr virus mRNA [50]. Although these interactions are generally interpreted as recruiting export factors to mature RNPs [51], recruitment of Nxf1 to the nascent transcript through retroviral or cellular RNA transport elements could, in principle, alter the recruitment or activity of splicing factors. Both the RNA binding activity and much of the known protein interaction network around Nxf1 are conserved with respect to the Saccharomyces homolog, Mex67p [11],[48],[52]. It is interesting in this context that in splicing-specific RNA profiling of yeast mutations with defects in mRNA production the expression profile of *MEX67*-deficient strains cluster with transcriptional elongation factors [53]. Altered elongation rate is thought to be one mechanism that can regulate alternative splicing [54] and recruitment of Nxf1 to elongating nascent transcript could in principle alter the assembly or kinetics of other factors on the elongating pre-mRNA.

The extension of suppressor activity to a wider class of insertional mutations has several practical implications. First, these results predict that *Nxf1^CAST^* should be able to modify other mutations that involve similar IAP insertions, for which new examples continue to be reported [43],[44],[55]. Indeed, the recent description of an IAP allele of *Pofut1* notes variable reduction of phenotype among F2 progeny in a cross to CAST/Ei, the strain from which the suppressing allele of *Nxf1* was derived [55]. The congenic *Nxf1^CAST^* stock we have developed should be a useful tool to allow in situ titration of gene expression from either spontaneous or engineered alleles involving such insertions. Second, the range of titration in each of the six cases we have examined is ∼1.5 to 2-fold and semi-dominant. This holds over a fairly broad range of mutational effects on gene expression, ranging from ∼2% and 4% of wild-type levels (unsuppressed and suppressed, respectively) for *Atcay^hes^* to 50% and 75% for *Eya1^BOR^*. Finally, our in vivo gene titration results across six different mutations suggests that for a wide range of loci and allele strengths, even modest recovery of function may have dramatic phenotypic benefits. This is strikingly true in the case of *Atcay^hes^*, where even a 2% increment of expression has a dramatic impact on behavioral phenotype (Videos S3 and S4). This implies that for Cayman ataxia, even a small amount of recovery in biochemical or cellular function would have substantial therapeutic benefit.

We have now demonstrated suppressor activity of the *Nxf1^CAST^* allele toward six different mutations with distinct biochemical and physiological properties in the mouse. To the best of our knowledge this is now the most broadly validated suppressor or modifier gene activity in this well-studied species.

## Materials and Methods

### Mice

Congenic C57BL/6J (B6)–*Nxf1^CAST^* mice were derived in our laboratory [4] and maintained by backcrossing to B6. Crosses described here were initiated with a stock at N19 or later backcross generation. C3H/HeJ–*Atrn^mgL^* and B6–*Mgrn^md^* were obtained from Dr. Teresa Gunn, Cornell University; mixed stock–*Ap3d1^mh2J^* and C3H–*Atcay^hes^* from Dr. Margit Burmeister, University of Michigan; B6–*Usp14^axJ^* from Dr. Scott Wilson, University of Alabama, Birmingham; and B6–*Mitf^mi-bw^* from Dr. Lynn Lamoreux, Texas A&M University. A/J and BALB/cJ were purchased from the Jackson Laboratory. Mice were maintained in specific pathogen-free conditions in accordance with protocols approved by the University of California at San Diego IACUC. Phenotypic comparisons were carried out using littermate pairs. Scores for behavioral phenotypes were assessed by at least 3 trained observers blinded to genotype. Videos of representative behaviors are available online as supporting information.

### DNA

Genotypes for *Nxf1* and each insertional mutation were determined by custom PCR assays for each locus. Conditions for PCR of full-length insertions were optimized using a commercial kit (MasterAmp Extra-Long PCR Kit, Epicentre) and primers in unique flanking sequences. DNA sequence analysis from the resulting PCR products used standard methods, as previously implemented in our laboratory [56] and assembled in Sequencher 4.8. Primers and PCR conditions are provided in the supporting information. Sequence alignments and neighbor-joining trees were performed in MUSCLE [57],[58] on the European Bioinformatics Institute web site (http://www.ebi.ac.uk/).

### RNA

Freshly dissected tissues were homogenized in Trizol reagent (Invitrogen) and processed for RNA according to the manufacturers instructions. Poly(A)^+^ RNA was purified by oligo(dT) cellulose chromatography. Northern blots were prepared from formaldehyde-agarose gels by capillary transfer to Hybond-N membranes and crosslinked by exposure to 2400 J UV light. Probes were prepared from cDNA fragments by random primer labeling. Hybridizations to each filter were quantified by phosphorimage analysis (Storm, Molecular Dynamics) and normalized to subsequent hybridization of *Gapd* to the same membrane as an internal control. Quantitative PCR assays were performed on total RNA. TaqMan assays for *Atrn* (Applied Biosystems, assays Mm00437738_m1 and Mm01270975_m1) and *Atcay* (Mm01172843_m1) were performed by the UCSD Center for AIDS Research Genomics Core Laboratory and normalized to a *Gapd* TaqMan assay. All other quantitative RT-PCR experiments were performed using intron-spanning primers that flank the inserted intron, detected by SYBR green fluorescence in a Bio-Rad CFX96 instrument, and quantified by the ΔΔCt method. Measurements were performed in triplicate for each sample. Samples to be compared were measured on the same plate during a single run. Custom primer sequences and conditions are provided as Tables S1, S2, S3, and S4 online.

### Protein

Freshly dissected tissues were homogenized in CelLytic M Cell Lysis reagent (Sigma #C2978) plus protease inhibitors and quantified using a bichromate assay (BCA, Pierce). Samples were subjected to SDS-PAGE and Western blotting onto Hybond-ECL membranes. Antibodies and dilutions used were goat anti-Ap3d1 (Rockland, 1∶1000), rabbit anti-Caytaxin/BNIP (Gift of Dr. Low Boon Chuan [59], 1∶5000), rabbit anti-Usp14 (Bethyl Laboratories, 1∶5000). Relative levels of immunoreactivity were quantified using infrared dye-coupled secondary antibodies (Rockland, 1∶10,000) on a LI-COR imager and normalized to rabbit anti-PITPβ [40] as an internal control that correlated with BCA-measured total protein.

### Statistics

Summary data are plotted in figures as mean values, with error bars indicating standard deviations. For variables with expected normal distributions, including quantitative PCR experiments and behavioral observations in which several observers rated performance against a calibrated scale, hypotheses were tested using paired or unpaired *t*-tests depending upon whether the underlying materials were from explicitly paired samples (e.g., matched littermates) or aggregates (e.g., sibs and cousins). For variables expected to have non-normal distributions across trials (including blotting procedures, in which normalization and scaling across experiments complicate the analysis, and paired samples for which some replicate pairs represent different ages or breeding designs) hypotheses were tested using a nonparametric Wilcoxon signed-ranks test applied to replicates of paired experimental measures. Statistical calculations were carried out in Microsoft Excel or SISA online, http://www.quantitativeskills.com/sisa/ [60] (t-tests) or using the VassarStats public web interface, http://faculty.vassar.edu/lowry/VassarStats.html (Wilcoxon tests).

## Supporting Information

Table S1 Genotype assays.
(0.02 MB XLS)

Table S2 qPCR assays.
(0.02 MB XLS)

Table S3 IAP insertions.
(0.01 MB XLS)

Table S4 Long PCR conditions.
(0.01 MB XLS)

Video S1 Video of behavioral phenotypes for *Usp14axJ* with *Nxf1B6*.
(0.26 MB MOV)

Video S2 Video of behavioral phenotypes for *Usp14axJ* with *Nxf1CAST*.
(0.58 MB MOV)

Video S3 Video of behavioral phenotypes for *Atcayhes* with *Nxf1B6*.
(1.20 MB MOV)

Video S4 Video of behavioral phenotypes for *Atcayhes* with *Nxf1CAST*.
(0.51 MB MOV)
